# Uterine cervical involvement of non‐Hodgkin lymphoma: Rare cause of postcoital bleeding

**DOI:** 10.1002/ccr3.4150

**Published:** 2021-05-04

**Authors:** Özer Birge, Mehmet Sait Bakır, Can Dinc, Ceyda Karadag, Ahmet Boduroglu, Tayup Simsek

**Affiliations:** ^1^ Department of Gynecology Obstetrics Nyala Sudan Turkey Research and Training Hospital Nyala Sudan; ^2^ Department of Gynecology Obstetrics Division of Gynecologic Oncology Akdeniz University Antalya Turkey; ^3^ Department of Pathology Akdeniz University Antalya Turkey

**Keywords:** B‐cell lymphoma, cervix uteri cancer, non‐Hodgkin lymphoma

## Abstract

It should be kept in mind that non‐Hodgkin lymphoma may involve uterine cervix and a multidisciplinary approach should be adopted.

## Graphical abstract

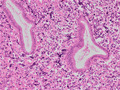

## 1 INTRODUCTION

The involvement of non‐Hodgkin lymphoma on female genital organs is extremely rare. When diagnosed, it can be confused with malignancies of the cervix uteri. A 40‐year‐old female patient who had presented with postcoital bleeding, dyspareunia, and vaginal discharge with a foul odor, preliminarily diagnosed with a cervical malignant mass, was diagnosed as non‐Hodgkin lymphoma with cervix uteri infiltration as a result of the pathological examination. PET‐CT revealed pathological involvement, and the medical oncology department administered chemotherapy. Cure was achieved in the patient with chemotherapy. In rare cases presenting with cervical uteri cancer, it should be considered that Non‐Hodgkin lymphoma may have systemic metastasis involving the cervix.

Non‐Hodgkin lymphoma is one of the most common cancer types diagnosed in men and women in developed countries.^1^ In women, malignant lymphomas comprise 3.5% of all malignant neoplasms. Most of these are NHL (73%). NHL is a heterogeneous group of malignancies originating from different differentiation stages of two distinct types of lymphocytes, namely B or T lymphocytes.^2^ The World Health Organization's (WHO) classification is currently the most used system.^3^ The 2016 revision of the WHO classification includes the definition of more than 50 NHL types and a few temporary types.^4^

Genital system involvement of non‐Hodgkin lymphoma can be seen in two ways. The first is localized lymphoma originating from the primary genital organ, and the second is genital organ involvement of systemic lymphoma, which is more common.^5^

Primary extranodal involvement of non‐Hodgkin lymphoma is rare. While the rate of primary extranodal disease is nearly 30% in Europe, it has been determined as 22%‐25% in America. Based on differences in geographical regions, this rate increases to nearly 50% in Middle Eastern and Asian countries.^6^, ^7^ The most common extranodal involvement areas of non‐Hodgkin lymphomas are the stomach, skin, small intestines, and the tonsils, but they can involve almost any system.^6^, ^7^ In particular, extranodal involvement may occur in many genitourinary regions such as the kidneys, testes, ovaries, ureters, bladder, uterus, and the cervix uteri in the form of infiltration of the lymphoma or primary lesions.^6^, ^7^, ^8^ Nearly 1% of lymphomas originating from the testicular region are primary. The clinical findings are similar regardless of whether it is primary or due to infiltration, and there are complaints of pressure feeling, pain, vaginal bleeding, hematuria, and organ failures secondary to obstruction, although rare, severe clinical conditions such as hypercalcemia, hyperuricemia, severe hypoglycemia, and renal failure can develop as complications secondary to non‐Hodgkin lymphomas.

## 2 CASE PRESENTATION

**FIGURE 1:**
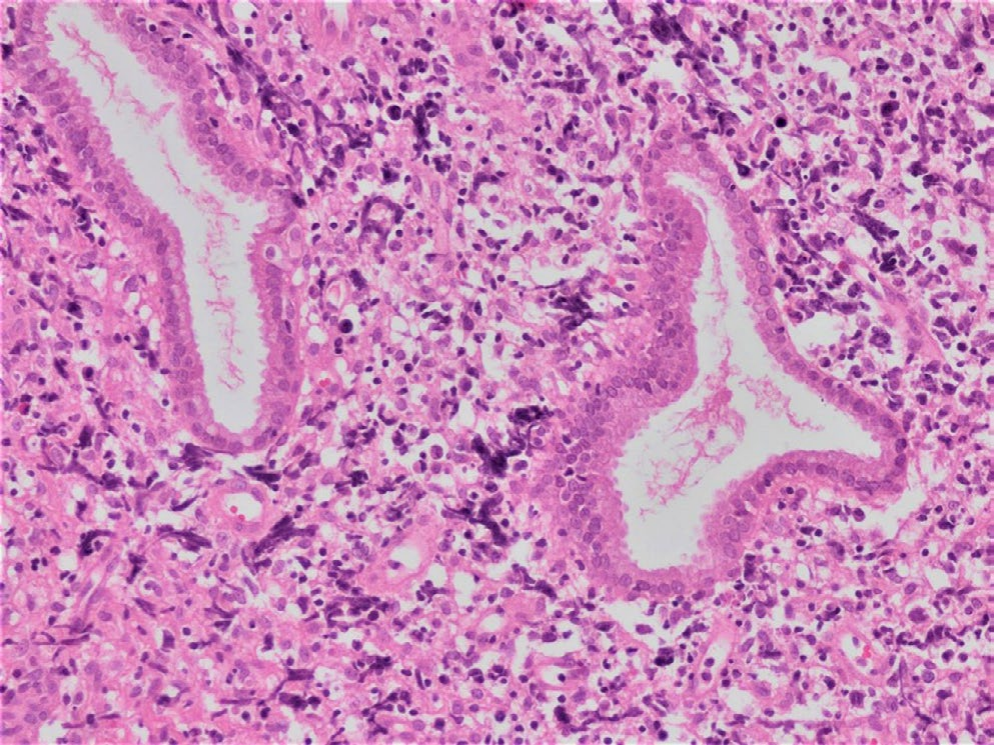
Diffuse neoplastic lymphoid infiltration composed of atypical neoplastic cells with large cytoplasm and hyperchromatic nuclei in the cervical stroma. Endocervical glands are entrapped in the neoplastic infiltration

**FIGURE 2:**
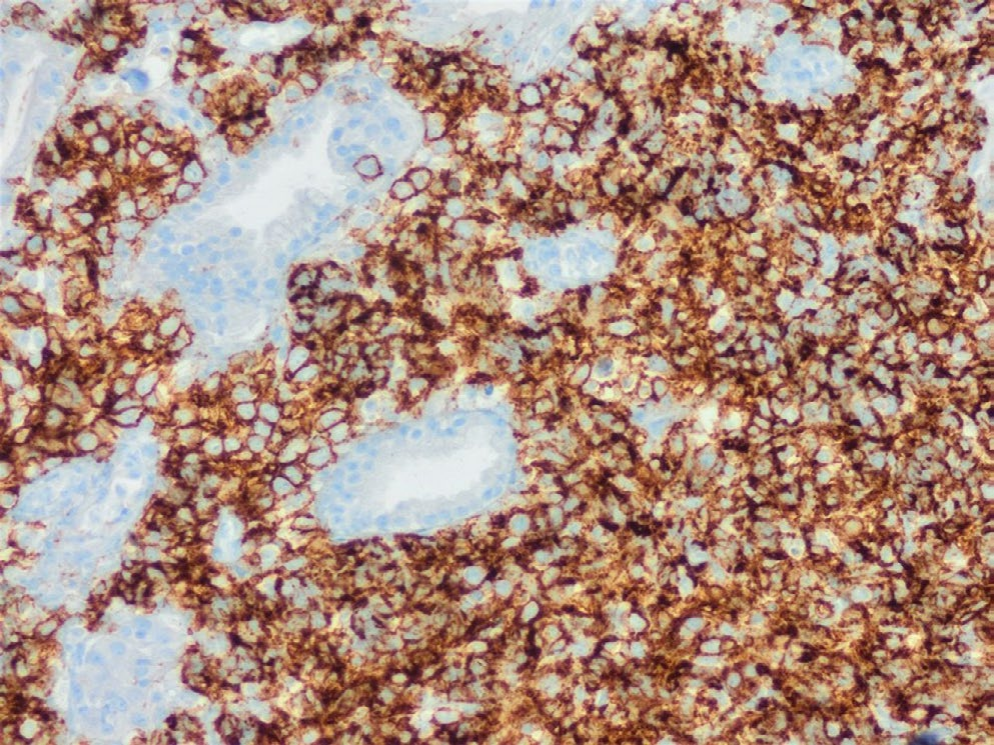
Neoplastic cells show diffuse and strong positive staining for CD20

After a mass was detected in the gynecological examination of the 40‐year‐old female patient who had presented with bleeding after sexual intercourse, dyspareunia, and watery vaginal discharge, and who had a history of 3 healthy normal vaginal births, a specimen for biopsy was obtained from the mass. The pathology was reported as cervical squamous type nonkeratinizing carcinoma, and the patient was referred to our tertiary hospital for advanced tests and treatment. The patient had no complaints other than postcoital bleeding, dyspareunia, and watery vaginal discharge with a foul odor. The gynecological examination revealed a normal vulva and vagina consistent with age; however, speculum examination revealed a massive lesion approximately 2 cm in size with irregular borders and fragile bleeding outside the transformation zone on the upper pole of the uterine cervix. The uterus was normal on ultrasound examination, and the ovaries and tubas had normal anatomical structure bilaterally. However, transvaginal ultrasonography revealed a normal uterine canal and a massive lesion of approximately 16 × 17 mm with irregular borders on the anterior surface of the uterine cervix that did not extend into the endocervical canal. A colposcopic examination was performed to obtain biopsy samples. The biopsies that had been carried out from the uterine cervical mass in another center also underwent the consultation of our hospital's pathology department. As a result of the pathological examination, CD 20‐positive high‐grade B‐cell lymphoma infiltration was determined. In the immunochemical examination of the preparations, the neoplastic cells were CD20, LCA and vimentin‐positive, and CD3, CEA, P63, CD56, and Pan‐CK negative (Figures 1 and 2). The positron emission tomography (PET‐CT) performed while waiting for the patient's pathology results revealed a right inferior jugular hypermetabolic millimetric lymph node with intense FDG uptake, hypometabolic in the center with hypermetabolic surroundings in the middle of the cervix, multiple hypermetabolic areas in the corpus uteri, multiple hypermetabolic lymph nodes in the abdomen, increased FDG uptake in the lesions close to the antral region of the small curvature of the stomach wall and fundus, intense FDG uptake in the liver areas that appeared hypodense, focal increased FDG uptake in the areas of filling defects in the bowel loops of the abdomen, and increased FDG uptake in the lytic areas observed in the left acetabulum, left femoral head, and the anterior end of the left first rib. After the imaging and the pathology studies, the medical oncology department administered systemic rituximab, cyclophosphamide, vincristine, and prednisone (R‐CHOP) chemotherapy and radiotherapy to the right humerus and the pelvic region. The control PET‐CT performed 4 months later revealed low FDG uptake in the lytic area observed in the right humerus head and a nonmetabolic millimetric nodule in the superior of the left lung lower lobe, and this was reported as findings consistent with the response to treatment. In the comparative PET‐CT repeated 6 months later, the lytic hypermetabolic area in the right humerus head remained unchanged, no pathological lymph node involvement was observed, and complete cure was seen to have been achieved after chemotherapy treatments. It was also observed that the cervix uteri lesion in the case had regressed and the complaints had resolved.

## 3 DISCUSSION

Non‐Hodgkin lymphoma is a malignancy of B, T, and natural killer (NK) cells that affect the lymphoid, hematopoietic, and other extranodal systems, arising from precursor or mature cells.^9^ Although the rate of increase has decreased recently, the incidence of Non‐Hodgkin lymphoma increases with the advance in age.^10^ The etiology of NHL is still being investigated, and until now, genetic, infectious, and various factors such as chemotherapy or radiotherapy have been considered as etiological factors.^11^ Hematological malignancies are rarely a primary gynecological problem. They usually occur as a result of systemic spread of tumor cells rather than primary tumors. Extranodal NHL often involves the gastrointestinal system and the bone marrow. Less than 1% of all extranodal NHLs affect the female genital system.^9^ NHL rarely infiltrates the female genital tract. Female genital tract lymphoma has an extremely low prevalence. Multicentric and interdepartmental joint studies are required to investigate these lymphomas.^6^ An interdisciplinary medical approach is required to diagnose, treat, and follow‐up this disease. Misdiagnosis of the primary tumor or infiltration of the female genital system should be avoided. Our case was also diagnosed with a cervix uteri tumor, and after a detailed evaluation, she was diagnosed with infiltration of the systemic malignant disease non‐Hodgkin lymphoma and cured with an appropriate treatment regimen. Furthermore, it is evident that morbidity and mortality would have increased if our case had been treated directly based on the specimens from another center.

In the study conducted by the International Workshop Group (IWG) in 2007, after analyzing the response criteria, FDG‐PET became the standard method of evaluating the response in both Hodgkin and non‐Hodgkin lymphomas.^10^ Due to the fact that NHLs are extremely sensitive to chemotherapy, the first treatment method is chemotherapy. CHOP‐R is the first treatment for this type of cancer. However, the follow‐up protocol and recurrence management are less known. In a review article, there are publications that do not recommend the addition of radiotherapy to chemotherapy in non‐Hodgkin lymphoma.^11^ It is recommended to add radiotherapy to chemotherapy, which is the main treatment, especially in patients with large masses.^12^

In final words, unnecessary genital organ losses can be prevented with multidisciplinary efforts in systemic lymphomas that mimic malignancies of the genital system and involve the female genital system and cause gynecological complaints. Furthermore, most importantly, it is seen that the best results can be obtained by making the definitive diagnosis promptly and starting treatment.

## CONFLICT OF INTEREST

None declared.

## AUTHOR CONTRIBUTIONS

ÖB and MSB: wrote the manuscript. CK, CD, AB, and TS: contributed to clinical follow‐up. TS: revised the manuscript.

## ETHICAL APPROVAL

None declared.

## INFORMED CONSENT

Informed consent was obtained from the patient to publish the case.

## Data Availability

All data analyzed in this case have been included in the article.
